# A snake toxin as a theranostic agent for the type 2 vasopressin receptor

**DOI:** 10.7150/thno.47485

**Published:** 2020-09-18

**Authors:** Laura Droctové, Manon Lancien, Vu Long Tran, Michaël Susset, Benoit Jego, Frederic Theodoro, Pascal Kessler, Gilles Mourier, Philippe Robin, Sékou Siramakan Diarra, Stefano Palea, Adrien Flahault, Amélia Chorfa, Maithé Corbani, Catherine Llorens-Cortes, Bernard Mouillac, Christiane Mendre, Alain Pruvost, Denis Servent, Charles Truillet, Nicolas Gilles

**Affiliations:** 1 Université Paris Saclay, CEA, INRAE, Département Médicaments et Technologies pour la Santé (DMTS), SIMoS, 91191 Gif-sur-Yvette, France.; 2 Université Paris-Saclay, CEA, CNRS, Inserm, BioMaps, Orsay, 91401, France.; 3 Université Paris Saclay, CEA, INRAE, Département Médicaments et Technologies pour la Santé (DMTS), SPI, 91191 Gif-sur-Yvette, France.; 4 Humana Biosciences, Prologue Biotech, 516 Rue Pierre et Marie Curie, 31670, Labège, France.; 5 Laboratory of Central Neuropeptides in the Regulation of Body Fluid Homeostasis and Cardiovascular Functions, Center for Interdisciplinary Research in Biology, INSERM, Unit U1050, Centre National de la Recherche Scientifique, Unite Mixte de Recherche 7241, Collège de France, Paris, France.; 6 IGF, Univ. Montpellier, CNRS, INSERM, Montpellier, France.; 7 INSERM, Unit U1050, Centre National de la Recherche Scientifique, Unite Mixte de Recherche 7241, Collège de France, Paris, France.

**Keywords:** Snake toxin, cancers, PK/PD, theranostic agent, PET imaging

## Abstract

**Rationale:** MQ1, a snake toxin which targets with high nanomolar affinity and absolute selectivity for the type 2 vasopressin receptor (V2R), is a drug candidate for renal diseases and a molecular probe for imaging cells or organs expressing V2R.

**Methods:** MQ1's pharmacological properties were characterized and applied to a rat model of hyponatremia. Its PK/PD parameters were determined as well as its therapeutic index. Fluorescently and radioactively labeled MQ1 were chemically synthesized and associated with moderate loss of affinity. MQ1's dynamic biodistribution was monitored by positron emission tomography. Confocal imaging was used to observe the labeling of three cancer cell lines.

**Results:** The inverse agonist property of MQ1 very efficiently prevented dDAVP-induced hyponatremia in rats with low nanomolar/kg doses and with a very large therapeutic index. PK (plasma MQ1 concentrations) and PD (diuresis) exhibited a parallel biphasic decrease. The dynamic biodistribution showed that MQ1 targets the kidneys and then exhibits a blood and kidney biphasic decrease. Whatever the approach used, we found a T1/2α between 0.9 and 3.8 h and a T1/2β between 25 and 46 h and demonstrated that the kidneys were able to retain MQ1. Finally, the presence of functional V2R expressed at the membrane of cancer cells was, for the first time, demonstrated with a specific fluorescent ligand.

**Conclusion:** As the most selective V2 binder, MQ1 is a new promising drug for aquaresis-related diseases and a molecular probe to visualize *in vitro* and *in vivo* V2R expressed physiologically or under pathological conditions.

## Introduction

Type 2 vasopressin receptor (V2R) is a G protein-coupled receptor (GPCR) encoded by the AVPR2 gene located on chromosome Xq28 1. It belongs to the vasopressin receptor family, like the V1a, V1b and oxytocin receptors. V2R is essentially expressed in the distal part of the nephron and in the collecting tubule of the kidneys and regulates water homeostasis under the control of arginine-vasopressin (AVP, 2). V2R, as many GPCRs, induces a pleiotropic action through both G protein-dependent and G protein-independent mechanisms, which are still not fully characterized. Once activated in the collecting duct, the V2R/Gαs pathway stimulates intracellular cAMP production, which activates protein kinase A to phosphorylate aquaporin 2, allowing its translocation from intracellular vesicles to the apical membrane via an intracellular calcium-dependent exocytosis mechanism. Further, water can go through aquaporin 2 at the apical membrane from the urine to the main cell before reaching the blood thanks to aquaporin 3 and 4 at the basolateral membrane 3. V2R also partners with β-arrestin for its internalization. Poorly recycled, V2R is associated with long-term activation thanks to an active complex AVP-V2R-β-arrestin 4. A third V2 pathway leads to the activation of ERK1/2 through metalloproteinase-mediated activation of the insulin-like growth factor receptor. This process is both Src- and β-arrestin-dependent 5.

Both loss- and gain-of-function variants of V2R are associated with human diseases and over 260 mutations have been reported to date (the Human Gene Mutation Database at the Institute of Medical Genetics, Cardiff) and recently reviewed 6. Some natural mutations, like R137C, R137L, I130N, F229V and L312S 7-10, are associated with higher V2R constitutive activities and cause the nephrogenic syndrome of inappropriate diuresis. In contrast, X-linked nephrogenic diabetes insipidus is a rare disease in male patients in which the ability to concentrate urine is reduced due to dysfunctional V2R trafficking, binding or function. Examples of V2R mutations are R137H, R181C, M311V and L312X 11.

Deficiency in AVP secretion is associated with hyponatremia with plasma sodium levels below 135 mmol/L in humans. It is associated with a number of diseases, including chronic heart failure, liver failure and chronic kidney disease, where it has been shown to be related to an increased risk of death. Drugs, including loop diuretics and thiazides, have also been associated with an increased risk of hyponatremia 12. Ectopic AVP secretion in lung or prostate cancers can also induce excessive circulating AVP levels, which can lead to hyponatremia 13. Finally, V2R is also the therapeutic target for the autosomal dominant form of polycystic kidney disease (ADPKD). Many therapeutic strategies have been developed to treat ADPKD, but only blockade of V2R is of proven efficacy in humans 14. Outside the kidney, endothelial cells express V2R where it participates in control of von Willebrand factor expression 15. In the inner ear, V2R probably participates in regulation of the lymph sac volume and is supposed to play a role in Meniere disease 16. In bone, V2R may contribute to homeostasis 17. Neuropeptides like AVP can be overexpressed in neuroendocrine tumors. V2R is present ectopically in human lung, breast, pancreatic, colorectal, and gastrointestinal tumors and its stimulation is related to anti-proliferative effects 13. Conversely, in renal tumors, its presence leads to proliferative effects 18.

Consequently, the potentially anti-metastatic properties of dDAVP, a specific V2R agonist, were used in clinical trials in patients with breast cancer (NCT01606072, 19) and in colorectal cancer patients with rectal bleeding (NCT01623206, 20).

Despite the crucial physiological and pathological importance of V2R, the therapeutic arsenal for this receptor is poor. Only one antagonist is principally used, named tolvaptan, a benzazepine-derived molecule, which acts as an antagonist on V2R with high affinity and moderate selectivity versus the three other vasopressin-sensitive receptors. Tolvaptan is used for treatment of hyponatremia and ADPKD 22, 23, but with many concerns due to its hepatotoxicity 24. Conversely, only one agonist molecule is used for central diabetes insipidus and enuresis, dDAVP (Minirin®), which is more stable and has a higher V2R selectivity than AVP 25.

We recently discovered the most selective V2R antagonist. Named mambaquaretin (MQ1), this peptide was isolated from mamba snake venom. MQ1 is a peptide of 57 residues cross- linked by three disulfide bonds adopting the Kunitz peptide structure 26. MQ1 displays high affinity for human V2R and no affinity at µM concentrations for 156 other GPCRs (including V1aR, V1bR and OTR). MQ1 is the first member of a new family of Kunitz peptides active on GPCR. Cell-based assays demonstrate that this peptide is a full competitive antagonist for the three pathways linked to the Gαs protein, the interaction with β-arrestin and activation of MAP kinase 26. When injected by the i.p. route in mice, MQ1 induces a pure aquaretic effect, meaning a loss of water without any loss of electrolytes. MQ1's efficacy against cystic kidney diseases was confirmed in CD1-*pcy* mice, which suffer from type 3 nephronophthisis, caused by a spontaneous missense mutation (T1841G) in the gene orthologous to human NPHP3 14.

In this present work, we deepened the molecular pharmacological characterization of MQ1, validated its use for hyponatremia and estimated its therapeutic index. Its pharmacokinetics, pharmacodynamics and biodistribution revealed how the toxin behaves *in vivo*. Finally, we used fluorescent MQ1 to label functional V2R expressed in cancer cells, thus opening the way to the development of a new diagnostic tool.

## Results

### Molecular pharmacological characterization of MQ1

**Figure 1:**
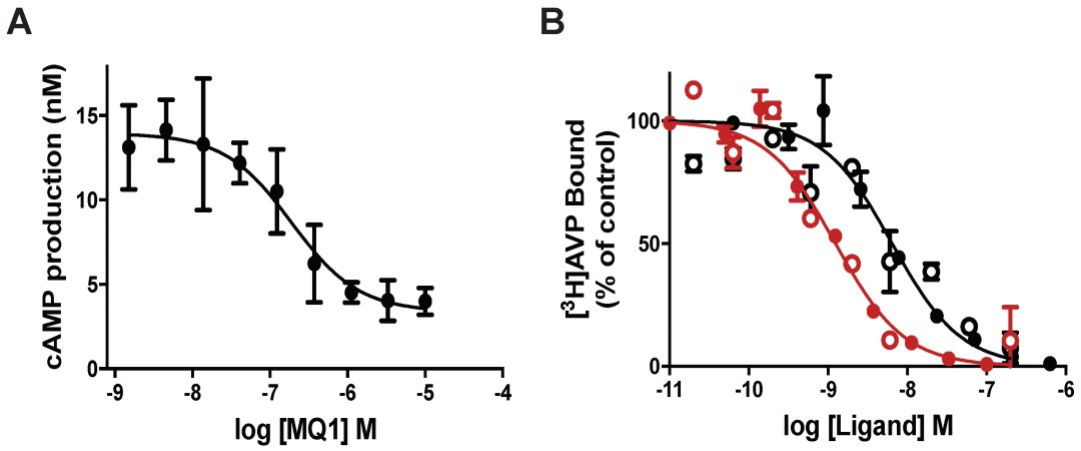
** (A)** Inverse agonist property of MQ1. cAMP concentration produced by the CHO cell line stably expressing hV2R as a function of MQ1 concentration. Representative curve, n=3, error bars are S.E.M. **(B)** Representative [^3^H]AVP competition curves by AVP (red) and MQ1 (black) on the CHO cell line stably expressing hV2R (open circle) or the COS cell line transiently expressing rV2R (closed circles). Representative curve, n=4-11, error bars are S.E.M.

We previously demonstrated that MQ1 has an antagonistic effect on cAMP production in cells expressing V2R naturally or heterologously 26. To deepen our understanding of MQ1's antagonistic property, we used a CHO cell line stably expressing high levels of human V2R, consequently leading to high basal cAMP production due to V2R's constitutive activity. Basal cAMP production by CHO cells depends on the number of cells in the plate: 3 to 5 nM cAMP with 5,000 cells/well to 13 to 15 nM with 20,000 cells/well. Using the latter condition, we applied various concentrations of chemically synthesized MQ1 (Figure S1) and evidenced a concentration-dependent decrease in cAMP production, with an IC50 of 167 ± 12 nM (Figure 1A), demonstrating its inverse agonistic property.

MQ1 and AVP affinities were determined on rat and human V2Rs. For hV2R, we confirmed the published affinities for AVP (0.71 nM, pKi of 9.15 ± 0.22, 27) and MQ1 (4.7 nM, pKi of 8.33 ± 0.20, 26). We transiently expressed rat V2R (rV2R) in COS cells and plotted concentration-dependent competition curves against tritiated arginine-vasopressin ([^3^H]AVP). AVP and MQ1 displayed rV2R affinities of 0.65 nM (pKi of 9.19 ± 0.28) and 7.7 nM (pKi of 8.14 ± 0.20), respectively (Figure 1B), values very similar to those found for hV2R.

### MQ1's effects in an experimental rat model of hyponatremia

**Figure 2:**
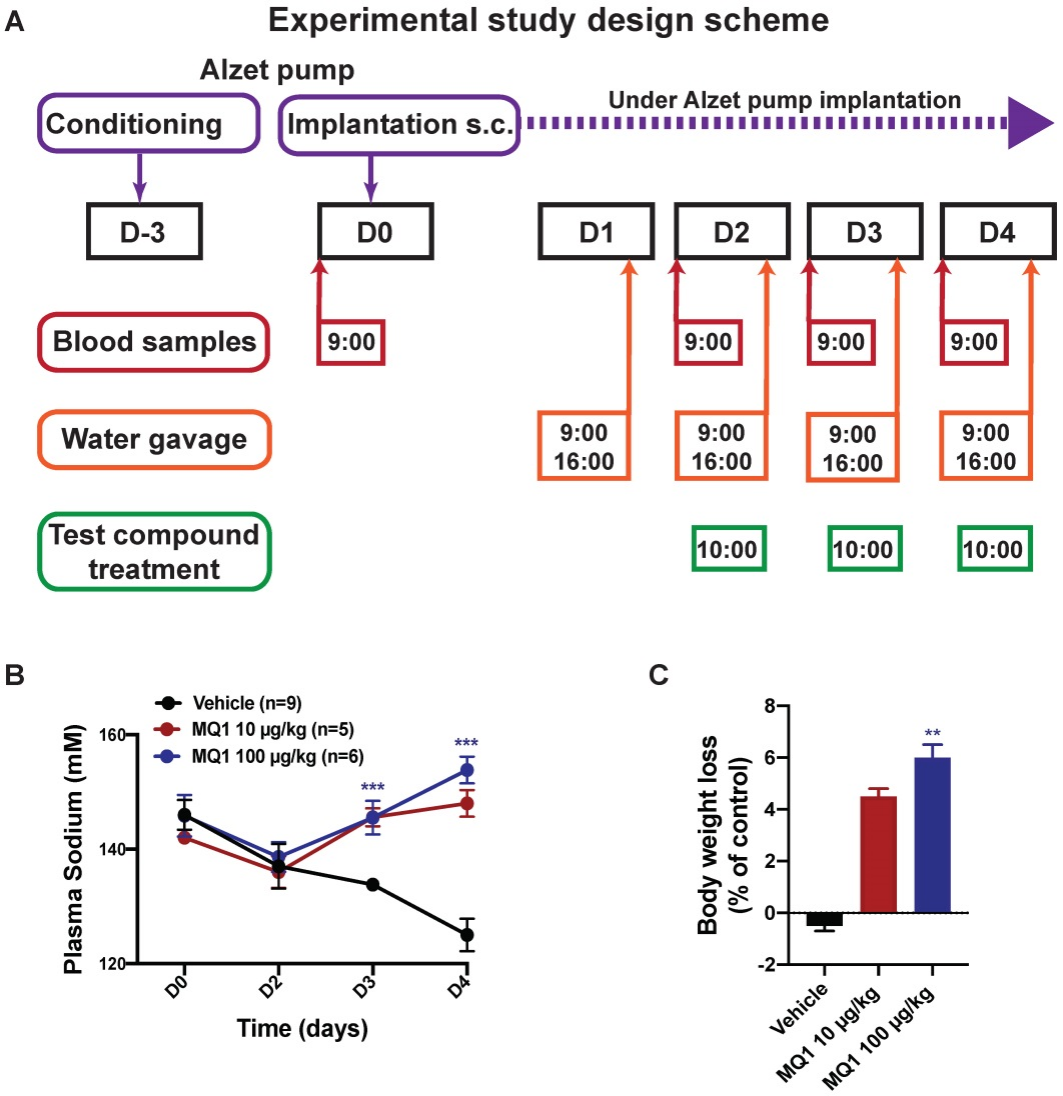
** (A)** Diagram of the experimental protocol. **(B)** Blood sodium as a function of time. Black circle, control. Red circle, 10 µg/kg (1.55 nmol/kg) MQ1.Blue circle, 100 µg/kg (15.5 nmol/kg) MQ1. **(C)** Rats bodies weights normalized to their own basal body weight.

We evaluated whether the aquaretic effect of MQ1 could reverse induced hyponatremia in a rat model of hyponatremia. Hyponatremia was induced in rats by continuous infusion of 10 ng/h of dDAVP and water gavage (Figure 2A). At day 0 (D0), the basal plasma sodium concentration was 145 ± 6 mM. At day 2 (D2), mean plasma sodium was significantly lower at 137 ± 7 mM (Figure 2B). In controls, plasma sodium decreased to 134 ± 1 mM and 125 ± 3 mM at D3 and D4, respectively. Following administration of 10 µg/kg (1.6 nmol/kg) or 100 µg/kg (16 nmol/kg) of MQ1, plasma sodium increased to 145 ± 3 mM and 146 ± 2 mM at D3 and 148 ± 2 mM and 154 ± 2 mM at D4, respectively (Figure 2B). These values were statistically different from the corresponding values in vehicle-treated rats at day 3 and day 4 (*P* < 0.0001), demonstrating the high efficacy of MQ1 in preventing dDAVP-induced hyponatremia. The concentration of 10 µg/kg increased plasma sodium by 12 mmol/l/24 h. This is considered as the highest acceptable correction rate in humans in order to avoid risk of osmotic demyelination 18. Rat weights were monitored during the experiment, each rat being its own reference. Referring to D0, control rat body weight was stable throughout the experiment (+0.36 ± 0.46%), while in MQ1-treated rats we noted a very slight decrease at D3 (-4.57 ± 0.63%) and D4 (-6.03 ± 0.95%, Figure 2C). We never noted any significant change in food intake during the experiments.

Our results reinforce MQ1 as a promising drug candidate by demonstrating its efficacy against hyponatremia, in addition to its capacity to reduce cysts in a PKD rodent model already described 26. We thus aimed to understand how this peptide behaves *in vivo* in terms of pharmacokinetics (PK), pharmacodynamics (PD) and biodistribution.

### MQ1 pharmacodynamics and pharmacokinetics

**Figure 3:**
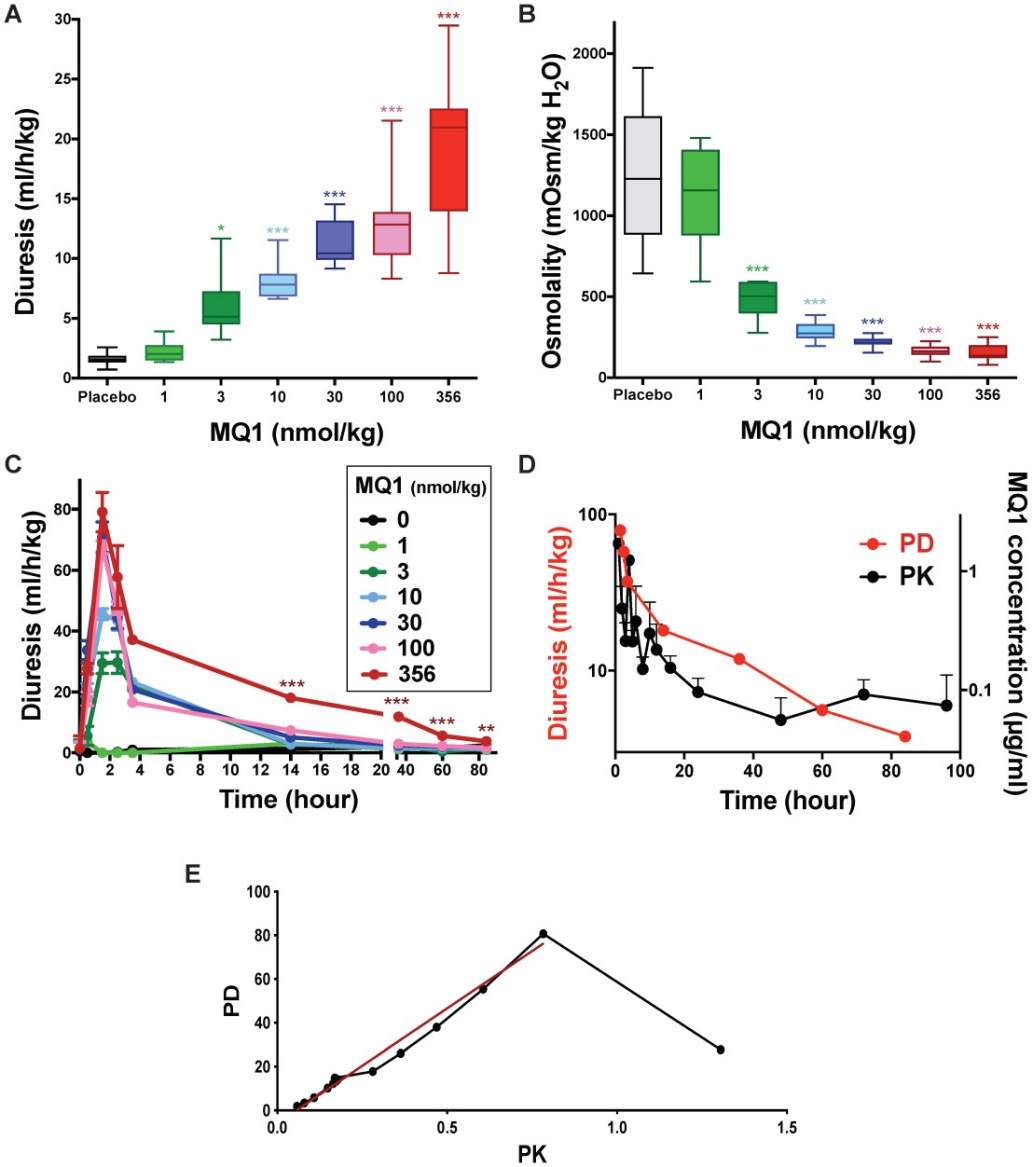
Rat diuresis **(A)** and urine osmolality **(B)** over a 24-hour period for various doses of MQ1. **(C)** Rat diuresis versus time for various doses of MQ1 administered by the i.p. route. Same color code for **(A), (B)** and **(C). (D)** Plot of diuresis (red and left axis) and MQ1 pharmacokinetics (black and right axis) for 356 nmol/kg MQ1. **(E)** Modeled pharmacokinetics vs pharmacodynamics relationship (black). Linear regression between 2- and 96-hour pharmacodynamics (red). PD= -5.6 + 102*PK. R^2^=0.98.

Control Sprague-Dawley rats urinated on average 1.56 mL/h/kg (0.72 to 2.59 mL/h/kg). When injected once with MQ1 at 1 nmol/kg i.p., diuresis and osmolality were statistically unchanged compared to control (Figure 3A and 3B). From doses of 3 to 356 nmol/kg, 24-h diuresis increased from 2.34 to 20.95 mL/h/kg (Figure 3A, Table 1) as osmolality decreased from 1158 to 136 mOsmol/kg H2O (Figure 3B, Table 1) inversely proportional to diuresis. Diuresis was maximal between the first and second h post-injection (pi), with volumes of 29.5, 45.6, 70.9, 67.6 and 79.1 mL/kg/h for the doses of 3, 10, 30, 100 and 356 nmol/kg, respectively (Figure 3C). Then, diuresis decreased mono-exponentially with a half-life of 3.7, 2.4, 2.4 and 1.2 h for the doses 3, 10, 30 and 100 nmol/kg, respectively. For the highest dose of 356 nmol/kg, the diuresis decreased quickly during the first 4 h and then lasted for four days. A bi-exponential analysis of this diuresis gave a fast half-life (T1/2α) of 1.2 h and a slow one (T1/2β) of 27.3 h (Figure 3D).

Blood samples were taken under heparin conditions between 1 and 96 h from rats injected i.p. with 356 nmol/kg. MQ1 was first purified from plasma using reverse-phase liquid chromatography (Table S1) before its quantification by mass analysis (Table S2). At 1 h post- injection, MQ1 was at its maximal plasma concentration of 1.9 µg/mL (141 nM). MQ1 pharmacokinetics are summarized in Table 2. Plasma MQ1 concentration could be fitted by two superimposed exponential terms, exhibiting half-lives of 3.8 and 31 hs (Figure 3D). These two phases accorded well with those found in pharmacodynamics for the same dose. The three last MQ1 concentrations at 48, 72 and 96 h were close to the limit of quantification and thus were not very precise (Table 3). After modeling of both PK and PD data based on their respective k values (Figure 3D, Table S3), a clear relationship was shown between plasma concentrations and diuresis values (Figure 3E). The high MQ1 concentration of the first h led to an increase in diuresis, but not yet a maximum effect corresponding to the decay for a correlation between PD and PK. Through this decay, the relationship between PK and PD was perfectly correlated (Figure 3E).

### *In vivo* behavior of MQ1 by noninvasive imaging: biodistribution and clearance

**Figure 4:**
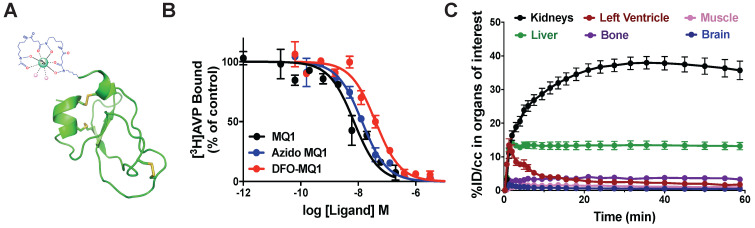
** (A)** Structural organization of DBO-MQ1. **(B)** Representative curves for the binding of MQ1 (black), 6-azidohexanoïc-MQ1 (blue) and DBO-MQ1 (red) to hV2R stably expressed in CHO cells. **(C)** Time activity curves of various organs within the first 60 minutes following intravenous injection in mice.

In order to assess the distribution and *in vivo* kinetic profiles, positron emission tomography (PET) is the most appropriate imaging modality thanks to its high sensitivity and dynamic imaging *in vivo*. We previously demonstrated that the MQ1 N-terminal is not essential for its affinity 26. We coupled a 6-azidohexanoic acid to the free amine group of the N-terminal arginine residue. After purification and oxidation of 6-azidohexanoic-MQ1 (Figure S1), a DFO-DBCO was cyclo-added to generate DFO-MQ1 (Figure 4A). 6-Azidohexanoic-MQ1 displayed the same affinity for V2R (7.9 nM; pKi of 8.10 ± 0.51) as wild-type MQ1, while DFO-MQ1 was 3.4 times less affine, with an affinity of 15.9 nM (pKi of 7.80 ± 0.37, Figure 4B). ^89^Zr tracer was attached via the DFO moiety to form ^89^Zr-DFO-MQ1 with a radiochemical yield higher than 95% after 60-min incubation at 37 °C (Figure S3).

^89^Zr-DFO-MQ1 *in vitro* stability tests in plasma and PBS for 7 days showed a high stability of the complex with less than 2% of free ^89^Zr or DFO-^89^Zr (Figure S2).

### Biodistribution

0.09 µg of ^89^Zr-DFO-MQ1 was injected into the tail vein of mice, which were then scanned during the first h following the injection. Different volumes of interest (VOIs) were drawn in order to determine the tissue time activity curves (TACs) of various organs: left ventricle (for ^89^Zr-DFO-MQ1 blood TAC input function, as described 28), kidneys, liver, muscle, bone and brain, expressed as the percentage of injected dose per volume (%ID/cc) as a function of time (Movie 1, Figure 4C). An extremely low uptake of less than 1% could be detected in muscle and brain, in accordance with the lack of V2R in these organs. Little uptake, between 4% at 1 h and 2% after 7 days, was observed in bone. It is well known that ^89^Zr (^89^Zr-DFO or ^89^Zr-oxalate) has a high affinity for bone 29. The uptake in the bone detected here is probably due to a small amount of free ^89^Zr present in ^89^Zr-DFO- MQ1, but not due to the presence of V2R in bone, which has been already reported 17.

^89^Zr-DFO-MQ1 was quickly distributed from blood to kidneys and liver, the two main organs of elimination. Kidney activity increased over time, reaching a plateau at 23 min compatible with specific accumulation due to its high V2R expression. Indeed, kidney ^89^Zr-DFO-MQ1 uptake (AUC of 1851% ID/cc.h) was two times higher than liver uptake (819% ID/cc.h, Table 3). The transfer rate between blood and kidneys (0.53 mL/min/g) calculated from the first min after injection of radiolabeled tracer was also higher than in the liver (0.32 mL/min/g, Table 3).

### *In vivo* elimination analysis over time

**Figure 5:**
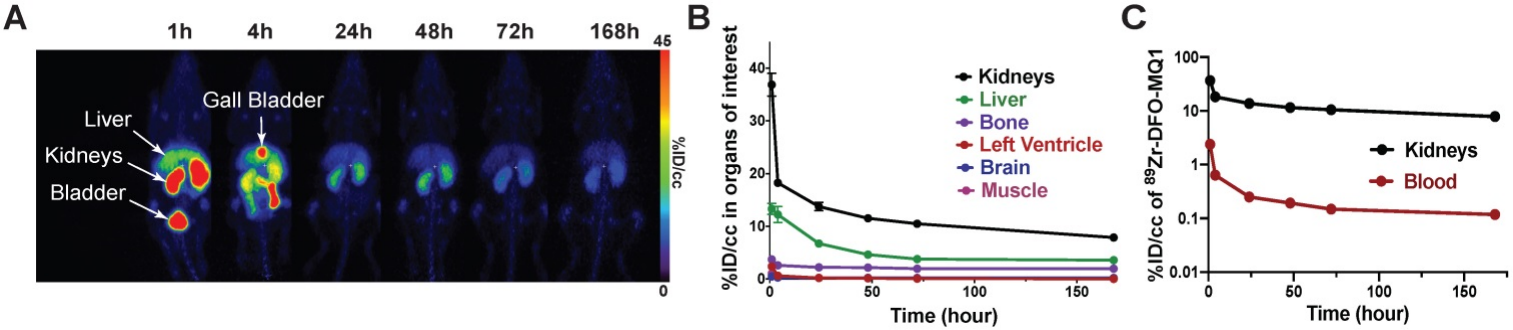
** (A)** Representative PET scans of healthy C57BL6 mice following the kinetics of tracer elimination within the seven days after intravenous injection of 89Zr-DFO-MQ1. **(B)** Biodistribution derived from (A). PET imaging by drawing VOI for various organs. **(C)** Blood and kidney activity curves over time.

Figure 5 represents the longitudinal behavior of ^89^Zr-DFO-MQ1 in the organism after intravenous injection in mice. In the liver, ^89^Zr-DFO-MQ1 taken up was quickly eliminated and decreased from 13 ± 2.5% ID/cc at 1 h post-injection to a residual plateau activity at 72 h post-injection (3.8 ± 0.35% ID/cc). DFO is a high-affinity Fe (III) chelator and the liver is the major site of iron storage, mainly through the complexation with ferritin. Residual radioactivity in the liver might be explained by some trapping of ^89^Zr in cells after transmetalation of ^89^Zr from DFO-MQ1 by iron. Metabolism of ^89^Zr in various forms should be studied to answer this question.

Elimination of blood ^89^Zr-DFO-MQ1 fits a bi-exponential curve and gave half-lives of 1.4±0.4 and 25±15 hours (Figure 5C). In the kidneys, ^89^Zr-DFO-MQ1 uptake decreased in the first hour from 37 ± 5.3% ID/cc at 1 h to 14 ± 1.9% ID/cc at 24 h. Thus, it takes six additional days to decline to 7.9 ± 1.5% ID/cc. Here again, a two-phases decay interpolation from the elimination curve provides half-lives of 0.9 ± 0.4 and 46 ± 12 h (Figure 5C), which are of the same order as those found for blood.

We investigated the *in vivo* stability of ^89^Zr-DFO-MQ1, in view of the potential metabolism of MQ1 over time or transmetalation of ^89^Zr released from the chelator. Blood was collected by heart puncture (n = 2) 1.5 h and 7 days post-injection (n = 6). iTLC analyses showed that there was no release of ^89^Zr or 89Zr-DFO during this period of time (Figure S2), indicating that all ^89^Zr detected by PET is attached to the peptide. HPLC analyses of blood at 7 days showed a single radioactive peak, which eluted at the same retention time as freshly prepared ^89^Zr-DFO-MQ1 (Figure S2). These two controls strongly suggest that ^89^Zr-DFO-MQ1 is highly stable over time and confirm that the radioactivity measured in mice corresponds to the active toxin.

Hydrophilic peptides below 25 kDa are rapidly eliminated from the kidneys through the glomeruli and are not reabsorbed through the renal tubule 30. The slow kidney elimination observed here comes from the high expression of V2R, which acts as a reservoir of MQ1.

### Therapeutic index

MQ1 as an *in vitro* and *in vivo* V2R inverse agonist can be seen as a drug candidate for ADPKD 26 and hyponatremia (this work). V2R antagonists reverse hyponatremia by increasing free water clearance. V2R antagonists also block cyst progression in ADPKD as V2R has been shown to be involved in cyst proliferation 31. For both diseases, blockade of V2R and, consequently, production of hypotonic urine are necessary.

**Figure 6:**
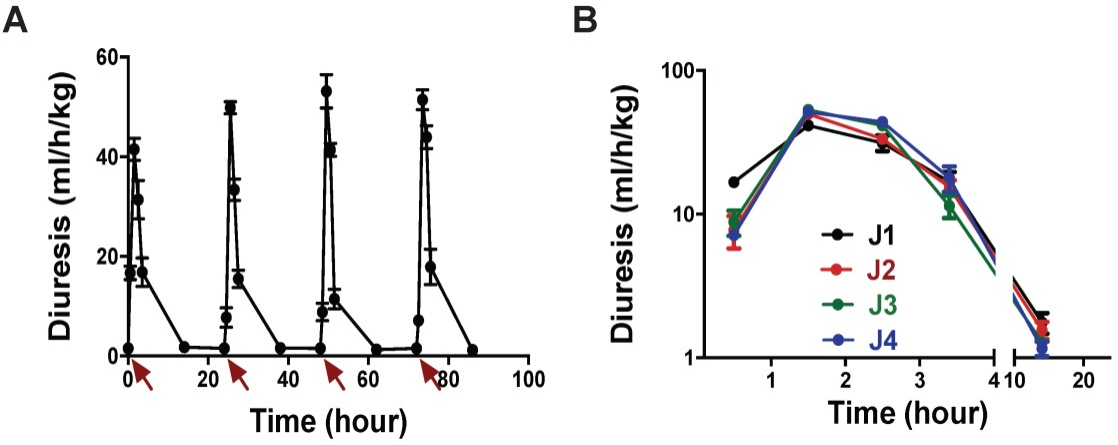
** (A)** Rat diuresis over four days. Red arrows indicate 3 nmol/kg MQ1 i.p. injections. **(B)** Superposition of rat diuresis monitored for four days.

Our dose-response curves (Figure 3A) and assays in the rat model of hyponatremia (Figure 2) indicated that the lowest dose of MQ1 producing hypotonic urine in rats was 3 nmol/kg given by the i.p. route. We injected this dose daily for 4 days and measured urine volume and osmolality. Diuresis increased 4.2-fold from 1.44 mL/h/kg in control rats to 6.0 mL/h/kg (Table 4), while osmolality decreased 4.3-fold from 1375 to an average of 321 mOsmol/kg, without any loss of electrolytes (Table 4). Diuresis always peaked between the first and second h following MQ1 administration (Figure 6A) and then decreased, with half-lives of 1.6, 1.3, 1.3 and 1.6 h for days 1 to 4 (Figure 6B).

We carefully observed animal behavior throughout the experiments. From the lowest to the highest tested dose, we never detected any observable toxic effects induced by MQ1 injections, such as prostration, spiky coat, appetite or weight loss. In conclusion, we may consider that MQ1 has a therapeutic index of about 100.

### MQ1 as a selective fluorescent probe for imaging cancer cells expressing V2R

Ectopic expression of AVP and its receptors has been reported in numerous cancers 32,33, with a potential anti-proliferative effect for V2R agonists in breast, pancreatic, colorectal and lung cancers 13,19,20,34-36 and an anti-proliferative effect for V2R antagonists in human renal carcinomas 33. In this context, MQ1 as a specific V2R antagonist constitutes an interesting probe for imaging cancer cells overexpressing V2R.

We generated fluorescent MQ1 probes by cyclo-adding a DBCO moiety to Cy5.5 or AFDye- 488 to 6-azidohexanoic-MQ1 (Figure S1). Both fluoro-MQ1s bind V2R with a slight loss of affinity compared to the wild-type toxin, with Kis of 24.5 nM (pKi of 7.61 ± 0.58) and 24.3 nM (pKi of 7.6 ± 0.5), respectively (Figure S3A). By confocal microscopy, we validated the concentration-dependent V2R labeling with increasing concentrations of AFDye-488-MQ1 (Figure S3B) and Cy5.5-MQ1 (Figure S3D). We also validated the molecular selectivity of the dyes by the absence of labeling for CHO cells stably expressing the three other vasopressin-oxytocin receptors (Figure S3C and E).

**Figure 7:**
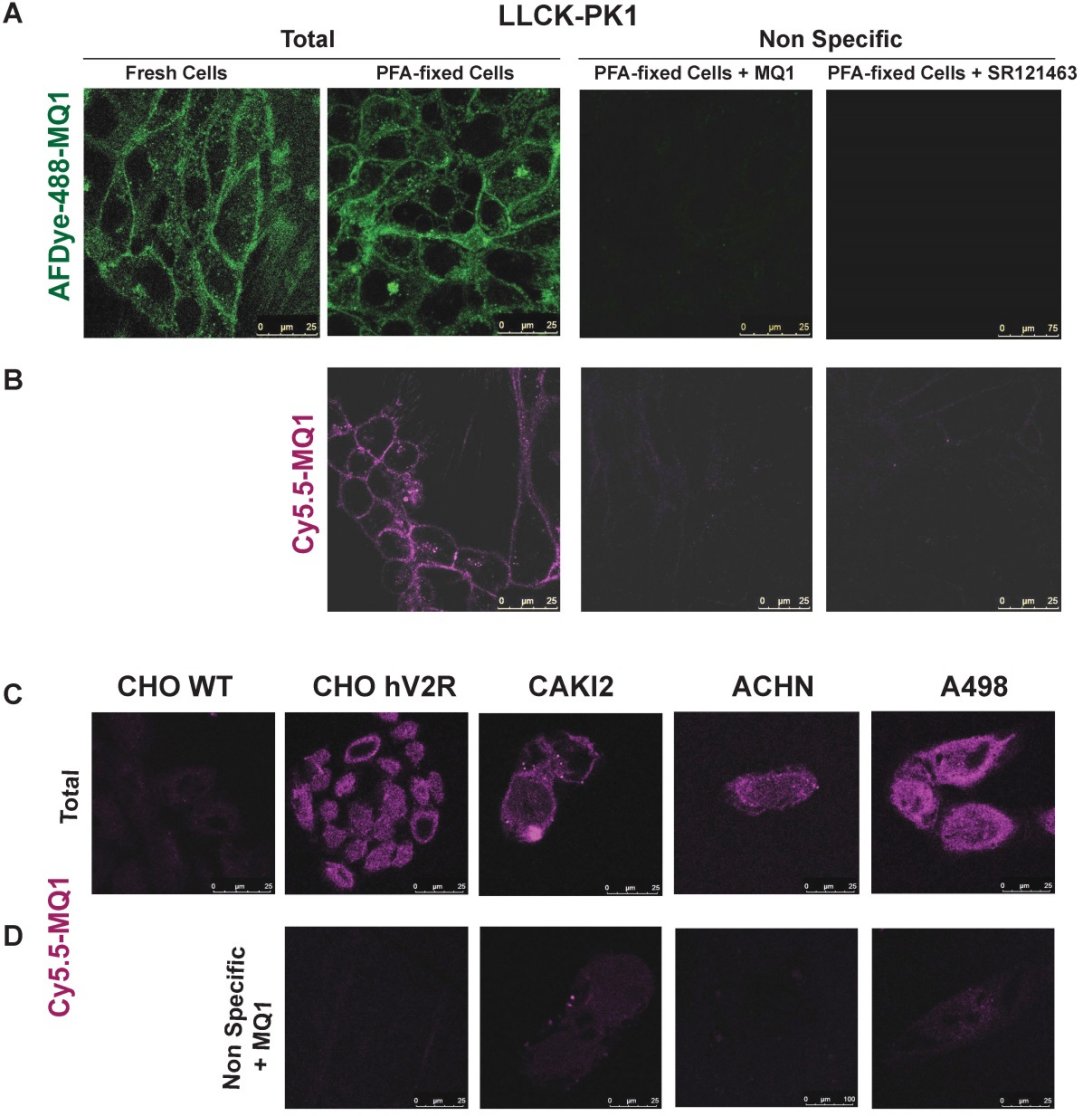
Labeling of endogenously expressed V2R in the LLC-PK1 cell line, in either fresh or PFA-fixed cells by **(A)** 100 nM of AFD-488-MQ1, **(B)** 100 nM of Cy5.5-MQ1. Specificity is determined by the last panels in the presence of either 3.4 µM MQ1 or 1 µM SR121463. **(C)** Cy5.5-MQ1 labeling at 100 nM in wild-type CHO cells (CHO WT), CHO cells stably expressing human V2R (CHO hV2R) and in renal cancer cell lines CAKI2, ACHN and A498 expressing hV2R. **(D)** Specificity is determined in the presence of 3.4 µM MQ1.

In cells endogenously expressing V2R, such as porcine kidney epithelial cells LLC-pk1, labeling of V2R with AFDye-488-MQ1 and Cy5.5-MQ1 (100 nM) was performed with either fresh or PFA-fixed cells (Figures 7A and B). In these conditions, the labeling was specific as 3.4 µM MQ1 or 1 µM SR121463, a non-peptide V2R antagonist 37, inhibited the labeling of the two dyes. Higher contrast and a lower background signal were obtained with Cy5.5- MQ1. In the pathophysiological context, three renal cancer cell lines (CAKI-2, ACHN and A498 38) known to express V2R were tested. Cy5.5-MQ1 intensely labeled non- permeabilized cells (Figure 7C, D). The presence of V2R, at the cell membrane level, fluctuated as a function of cell density and the number of cell divisions, whatever the cell line used. Being a large hydrophilic peptide, MQ1 cannot cross the cell membrane, meaning that only functional V2R could be detected. These preliminary results demonstrate that MQ1 could become a novel and promising functional V2R probe *in vivo*. To our knowledge, this is the first specific ligand labeling of V2R in non-permeabilized cancer cells.

## Discussion

Animal venom toxins target mainly enzymes and ionic channels involved in the control of the hemostatic, nervous, and cardiovascular systems 39-43. MQ1 is unique as the first member of a Kunitz-fold toxin active on a G protein-coupled receptor 26. Kunitz peptides are present in many snakes and various others phyla such as arthropods, insects, and cnidarians 44. These toxins are known to principally block potassium channels 45 and/or to inhibit serine proteases 46 or both 47. MQ1 is associated with a new and unexpected function regarding V2R, and thus expands the repertoire of activities associated with the Kunitz fold.

Vasopressin regulates water homeostasis in the body by modulating urine concentration. It binds to the V2R receptor present in the principal cells of the kidney collecting tubules and activates the Gs/cAMP/phosphokinase A (PKA) signaling pathway. Then, activated PKA phosphorylates serine 256 of cytosolic aquaporin 2 (AQP2), which in turn translocates under AQP2-bearing vesicles to the apical plasma membrane leading to water reabsorption from urine into principal cells. Then, water reaches the plasma through basolateral membrane aquaporins 3 and 4, thus increasing blood volume and decreasing plasma osmolarity 48. Targeting the vasopressin-V2R-aquaporin axis can provide therapeutic benefits in water balance disorders. Tolvaptan, a V2R antagonist, is used for its ability to inhibit cAMP in principal cells and was shown to inhibit aquaporin traffic and function leading to diuresis 49. As described earlier 26 and in the present study, MQ1 is a new antagonist/aquaretic V2R ligand which may function like tolvaptan by blocking V2R/aquaporin-related effects, leading to the absence of water reabsorption evidenced by a diuretic effect.

We show here that MQ1, as an inverse agonist, is very efficient in V2R blockade and aquaretic *in vivo* effects as it also reduces V2R constitutive activities. This approach can be applied to ADPKD 26 and hyponatremia (this work). Hyponatremia is the most common disorder of body fluid and electrolyte balance. Severe hyponatremia may lead to brain edema leading to neurological symptoms or death, although this outcome is rare 18. Mostly, hyponatremia is chronic and rather asymptomatic, but is associated with increased mortality in hospitalized patients 50. Therefore, acutely hyponatremic patients need to be treated rapidly. At a dose as small as 10 µg/kg (1.6 nmoles/kg), MQ1 prevented dDAVP-induced hyponatremia in rats. Vaptans are the only V2R-targeting drugs approved for treatment of hyponatremia, but their use is extremely limited due to their hepatotoxicity. ADPKD is considered as the fourth leading cause of end-stage renal disease and the most prevalent genetic kidney disease. It has an incidence from 1 in 1000 to 1 in 400 individuals, and affects over 12 million people worldwide. Here again, only tolvaptan has reached the market and its use is subject to many concerns 24. MQ1 constitutes a new paradigm in the treatment of both hyponatremia and ADPKD and a better understanding of its *in vivo* behavior is essential to develop effective therapeutic and diagnostic tools.

We used complementary technologies in rats and mice to elucidate how the toxin spreads in the organism and how it is eliminated. Once injected, MQ1 targets the kidney, where it accumulates for many days. This was shown by clear longstanding diuresis (PD) and persistent kidney labeling (PET imaging). In contrast to the *in vitro* closed and stable conditions that allow precise quantification of the parameters of a drug binding to its target (K_d_, K_on_, K_off_), the *in vivo* situation is an open and unstable environment. Fifteen years ago, some authors proposed that the most important pharmacological parameter in predicting *in vivo* activities is K_off_, as pharmacological activity depends on the binding of the drug to its intended target and will usually only persist while the drug remains bound 51. If we apply this theory, the *in vitro* MQ1/V2R half-time should be between 25.7 and 46.5 hrs (K_off_ between 0.015 and 0.027 h^-1^, for simplification we neglected the small proportion of blood MQ1 likely to bind the kidney). With a such slow K_off_, the incubation time to reach equilibrium in competition binding assays should be more than 5 days 52. Competition between ^3^HAVP and MQ1 is stable after 2 h of incubation (data not shown), demonstrating that MQ1/V2R K_off_ is much faster. Another theory advances that K_on_ is more important than K_off_ in predicting *in vivo* efficacy 53. In this theory, the notion of *in vivo* re-binding describes how a drug, which dissociates from its target, can re-bind immediately thanks to a fast K_on_ and a high receptor concentration of the target organ. Kidneys express a high density of V2R, concentrated in the distal tubule and the principal cells of the collecting duct. One hypothesis could be that MQ1 re-binds to neighboring receptors thereby resulting in a very long macro residence time associated with lengthy effects.

Aprotinin and bikunin are two Kunitz peptides. Aprotinin is used clinically in acute pancreatitis, shock syndromes and hyperfibrinolytic hemorrhage. Bikunin is a protease Kunitz inhibitor that inhibits the invasiveness of tumor cells of various histological origins. Aprotinin 54 and bikunin 55 have similar T_1/2α_ of 0.7 and 0.85 h and T_1/2β_ of 7 and 6.4 h, respectively. Aprotinin, bikunin and MQ1 have a similar T_1/2α_, reflecting the same mode of elimination, which is considered to be principally renal for aprotinin and bikunin. However, neither aprotinin nor bikunin possesses an *in vivo* target reservoir as MQ1 does. This may explain their much shorter T_1/2β_ and reinforce the hypothesis of re-binding for MQ1. More *in vitro* and *in vivo* experiments will be necessary to explore in depth this hypothesis and uncommon *in vivo* behavior.

Because of its size (6372 Da) and solubility, MQ1 is most probably eliminated by glomerular filtration. The fact that the gall bladder is labeled at 4 h post-injection suggests that hepatic elimination may also occur, at least during the first h. Liver peptide metabolism is not well documented and this study opens the door to an interesting question about the behavior of MQ1 in the liver. A complete metabolism study of MQ1 should be done to fully understand its hepatic and renal elimination.

The intrinsic qualities needed to develop a good *in vivo* contrast agent are high molecular selectivity so as to label only the selected target, a high affinity, so that a low dose of the tracer can be injected, quick blood elimination to reduce background noise and long-term residence in the targeted organ. MQ1 has these qualities. As a peptide, MQ1 is simple to engineer. 6-Azidohexanoic-MQ1, which is compatible with click chemistry, can be labeled by a large variety of contrast agents. We exemplified this statement by generating the first PET agent specific to V2R and two highly efficient specific fluorescent probes, *in vitro*, to label native V2R ectopically expressed in cancer cell lines. Expression of V2R on cancer cells was also validated at the mRNA level and by immunostaining of fixed tissues 33, 34, 38, but the antibody's specificity and capacity to bind to fresh cells is not documented, unlike in our study in which fluorescent MQ1 bound native, functional, extracellular V2R with high affinity and selectivity. ^99^mTc-AVP was generated and proved able to label V2R *in vitro* 56. This tool may be of high interest *in vivo*, but with two limitations. First, AVP is not selective and will also label V1aR, V1bR and OTR and, second, AVP in rats has a shorter half-life, with a T_1/2α_ of 0.9 min and a T_1/2β_ of 8 min 57.

## Conclusions

Nature is still a source of unexpected activities. Animal venoms are virgin territory as less than 0.1% of their toxins have been studied and many other unpredictable activities are waiting to be discovered. Like many venom-derived peptides, MQ1 has a high affinity and selectivity for V2R and is devoid of any toxic effect. MQ1, as a natural peptide toxin, has been continuously selected and highly refined by the evolutionary process and has pharmacological properties that are highly valuable in the context of human use and drug development. MQ1 constitutes a new paradigm for many V2R-related biological questions and we strongly believe in its use as a therapeutic drug candidate and diagnostic tool.

## Methods

### Materials

Unless mentioned otherwise, all chemicals were from Sigma-Aldrich. AVP was from Bachem (Bubendorf, Switzerland), [^3^H]AVP was from PerkinElmer (Courtaboeuf, France), and DFO-DBCO was from Macrocyclics (Plano, USA). Fmoc-amino acids, Fmoc-pseudoproline dipeptides, and 2-(6-chloro-1-H-benzotriazole-1-yl)-1,1,3,3,-tetramethylaminium hexafluorophosphate (HCTU) were from Activotec (Cambridge, UK). The cAMP assay kit was from Cisbio (Marcoule, France). Cy5.5-DBCO and AFDye-488-DBCO were from Click Chemistry Tools (Scottsdale, AZ 85260, USA).

### Chemical synthesis of MQs

MQ1 was synthesized using a Gyros Protein Technologies, Inc Prelude synthesizer at a 12.5 µmol scale, and then deprotected, purified and folded as described 26. A unique batch of MQs of purity higher than 95% was used for all experiments. 6-Azidohexanoic was coupled to the resin after the automated synthesis of MQ1 and the deprotection of the N-terminal amine function. 6-Azidohexanoic (2 eq) was coupled twice for 60 min with the coupling agent HCTU (1.9 eq) in the presence of 2 eq of diisopropylethylamine. 6-Azidohexanoic-MQ1 was cleaved from the resin, purified and oxidized, as for MQ1. 10 eq of DFO-DBCO (p-isothiocyanatobenzyldesferrioxamine-diarylbicyclooctyne, dissolved in 200 µL of DMF) or of Cy5.5-DBCO (dissolved in 200 µL HEPES buffer) or of AFDye-488-DBCO (dissolved in 200 µL of HEPES buffer) was mixed with 0.3 µmol of 6-azidohexanoic-MQ1 dissolved in HEPES buffer (200 µL, pH 7.4), left overnight at room temperature and purified by HPLC.

### Cell culture and cAMP assays

COS cells were cultured in Dulbecco's MEM medium supplemented with 10% FBS and transfected for the transient expression of rV2R. 48 hs after transfection, cells were centrifuged and membranes were prepared as described 26. The CHO cell line stably expressing hV2R was cultured as described 26, seeded at 20,000 cells per well in a 384-well plate and incubated with various concentrations of MQ1 for 30 min at 37 °C. The reaction was stopped by the addition of lysis buffer from the cAMP Dynamic 2 kit (Cisbio International), as described 26.

### Binding assays

Binding experiments were performed using 1 nM [^3^H]AVP in a 100-µL reaction mixture as described 26. Competition binding data were fitted to a one-site inhibition mass action curve using GraphPad Prism (San Diego, USA) and IC50 values were converted to Ki using 1.1 nM as [^3^H]AVP Kd 58. Data represent at least three independent experiments performed in duplicate and are presented as mean ± SD.

### Rat model of hyponatremia

Specific pathogen-free adult male Sprague-Dawley rats (body weight between 445 and 525 grams) were from Janvier laboratories (Le Genest St. Isle, 53941, Saint Berthevin Cedex, France) and acclimatized to the animal house conditions for 1 week. We established an experimental protocol derived from a previous paper 59 to develop a rat model of hyponatremia, approved by the French Ministry of Education and Research (No. 18604-2019010915104191). A stock 2.2 mg/mL solution of desmopressin (dDAVP) was prepared by dissolving 5 mg of drug in physiological saline. dDAVP was administered using subcutaneous ALZET osmotic mini-pumps (model 2002, DURECT Corporation, Cupertino, CA95014, USA) previously filled with a solution of dDAVP (pumping rate of 0.47±0.02 µL/h and mean fill volume of 233.6±4.7 µL). The dose of dDAVP (10 ng/h) was determined in preliminary experiments. Buprenorphine (Centravet, 03120 Lapalisse, France) was administered once a day at a dose of 0.02 mg/kg, s.c. from day 0 to day 3 in order to suppress post-operative pain. Water gavage (30 mL/kg) was performed 2 times per day (at 9 am and 4 pm) in the first 3 days following ALZET pump implantation, and at 9 am at day 4. Body weight was recorded every day in order to adjust for the volume of water to be administered. Rats had free access to standard rat chow, as well as water in their cages. MQ1, dissolved in physiological saline, was administered at 10 am on days 2-3-4 at 10 or 100 µg/kg (s.c. route, 0.8 mL/kg). On days 0, 2, 3 and 4, at 9 am, 400 µL of blood was collected from isoflurane- anesthetized rats from the tail vein with lithium heparinate (Sanofi-Aventis, Gentilly, France). Samples were centrifuged (4 °C, 2000 g, 5 min). Sodium was quantified by the Central Laboratory of the ENVT, Toulouse, France, on a VITROS 250/350/950/ 5, 1 FS, 4600 and integrated system VITROS 5600 (Ortho-Clinical Diagnostics, Buckinghamshire, United Kingdom). Multiple factor ANOVA followed by Tukey's test were used to test for significance. A *P* value < 0.05 was considered significant.

### Pharmacodynamics analysis

Sprague-Dawley rats are aged between 6 and 12 weeks, between 237 g and 545 g and were use up to 3 times. They were acclimated in metabolic cages (Techniplast France, Lyon, France) for two days, with food and water ad libitum, before being i.p. injected (fixed volume of 1 mL) with various doses of MQ1 (0 to 356 nmol/kg dissolved in 0.9% NaCl, French agreement number 2015082111349702v1). Urine was collected at various times, and centrifuged for 30 min at 20,800 g. Urine osmolality was determined with an osmometer (Knauer, Berlin, Germany). For the pharmacokinetic experiments, a catheter was implanted in the rat jugular vein five days before the experiment. Blood samples of 400 μl were taken at various times under heparin conditions. Blood was centrifuged at 2000 g for 5 min at 4 °C and plasma was frozen at -20 °C.

### Pharmacokinetic analysis

Sample preparation is described in Table S1. Concentrations of MQ1 in rat plasma were determined using an UPLC-MS/MS system comprising a UPLC Nexera series 30 (Shimadzu, Noisiel, France) coupled to a Quantum Ultra mass spectrometer (Thermo Fisher Scientific, Les Ulis, France). Chromatographic separation was achieved with an Acquity BEH C18 column (2.1 × 50 mm, 1.7 µm, 130 Å pore size, Waters, St Quentin en Yvelines, France). The mobile phase consisted of 0.1% formic acid in water (A) and 0.1% formic acid in acetonitrile (B) running in a gradient mode from B 2% to B 100% in 3 min. Total run time was 6 min and MQ1 retention time was around 2.12 min. Calibration standards were prepared by adding MQ1 to control plasma (EDTA-3K) obtained from Sprague-Dawley rats in the range 0.06 to 5 µg/mL. The mass spectrometer was operated in positive ESI and MRM mode. Details of the monitored transitions are given in Table S2. MQ1 concentrations of each rat (n = 3) were used to calculate the PK parameters by non-compartmental analysis using Kinetica software, version 5.1 SP1 (Thermo Fisher Scientific, Les Ulis, France). A model was selected based on the extravascular (i.p.) route of administration. For the i.p. route, concentration at time zero was assumed to be zero. For each rat, the first concentration below the LLOQ was set at LLOQ/2 for calculation of PK parameters. The area under the plasma concentration versus time curve (AUC) was calculated using the linear trapezoidal method (linear interpolation). When appropriate, the terminal elimination phase of the PK profile was estimated based on the best fit (r^2^) using at least the last six observed concentrations. Then, a mean PK profile was established and, based on non-compartmental analysis, two *k* values were determined as 0.25572 and 0.01310 for the distribution and elimination phases, respectively. The same approach was used to model diuresis over time and two k values were obtained as 0.37774 and 0.02326. Lastly, the PK/PD correlation was achieved after calculating concentrations and diuresis volumes over time based on the equation y = A_0_*e^-kt^ where *A_0_* was the computed concentration or diuresis volume at the origin of each phase.

### Biodistribution in mice

#### Radiolabeling of MQ1 with ^89^Zr

^89^Zr was supplied as Zr4+ in 1.0 M oxalic acid (PerkinElmer, Iba molecular, Netherlands). It was neutralized with 2.0 M Na2CO3 and its pH was adjusted to 7 with HEPES buffer. DFO-MQ1 (1 mg/mL, pH 7.0, HEPES 0.1 M) was radiolabeled with ^89^Zr (90 MBq) at 37 °C for 60 min and filtered through a PD-10 column (GE Healthcare Life Sciences, desalting columns, Sephadex G-25 medium) using gentisic acid solution. The radiochemical yield and purity were determined using instant thin-layer chromatography (ITLC-SG, Agilent, Santa Clare, USA), with citric acid (pH 4.9-5.1) as mobile phase. ITLC separated two phases that were analyzed using a gamma counter (Cobra II, Canberra-Packard, Schwadorf, Austria): (i) the migration phase with the free ^89^Zr or 89Zr-DFO and (ii) the non- migration phase with toxin (Figure S2). ITLC migration was confirmed by radio-TLC detection and radio-HPLC (Dionex, HPLC coupled with Flow Scintillation Analyzer- Flo- one, Packard). Separation was achieved using a Symmetry C18 Column, 100 Å, 5 µm, 3.9 mm × 150 mm (Waters). The mobile phase consisted of 0.1% trifluoroacetic acid in water (solvent A) and acetonitrile (solvent B). A linear gradient from 10% to 40% of solvent B over 30 min was applied to the column at a flow rate of 1 mL/min. The retention time of MQ1 was 20 min. The final specific activity was determined to be 1.06±0.23 ng/µCi. Radio-HPLC was developed to characterize the intact fraction of MQ1 in the blood (Figure S2).

#### ^89^Zr-DFO-MQ1 stability

For assessment of the *in vitro* stability of ^89^Zr-DFO-MQ1, two sets of experiments were performed. In a first set, labeled MQ1 was stored at 37 °C in phosphate buffered saline (PBS) for 7 days. The final activity concentration was between 0.6 and 1.2 MBq/mL. At various time points, aliquots were taken and analyzed by ITLC. In a second set, purified radiolabeled MQ1 was added to bovine serum at a final concentration of the radiolabeled conjugates of 0.6-1.2 MBq/mL. The samples were incubated at 37 °C in a CO2- enriched atmosphere (5% CO2). At various time points, aliquots were taken and analyzed by iTLC.

For assessment of *in vivo* stability, blood samples 7 days after injection of ^89^Zr-DFO-MQ1 were immediately subjected to centrifugation at 2300 g for 15 min at 4 °C, and the plasma supernatants were collected, aliquoted and studied. Activity from the blood samples was resolved by ITLC, which separated the whole ^89^Zr-DFO-MQ1 fraction from the free ^89^Zr or ^89^Zr-DFO. Each experiment was carried out in triplicate. For the blood analyses with HPLC, blood samples in heparin tubes were centrifuged and the plasma was separated. Samples were highly diluted before injection into the HPLC.

#### Animal experiments

Experiments in mice (C57BL/6JRj, Janvier Labs) were conducted according to European directive 2010/63/EU and its transposition into French law (Décret n° 2013-118). Animal experiments were conducted at the CEA-SHFJ imaging facility (authorization D91-471-105/ethics committee No. 44). Five-week-old male C57BL6 mice were from Charles River.

#### PET imaging session protocol with the radiolabeled constructs

^89^Zr-DFO-MB1 was administered via tail vein injection in < 150 µL saline solution. Mice were anesthetized and imaged with a Siemens Inveons (Siemens, USA) by small animal PET/CT or PET at dedicated time points post-injection. For each mouse, 60-min dynamic scans were performed just after injection of ^89^Zr-DFO-MQ1 (0.13 MBq/g). A 30-min scan was then done at 4 h, 24 h, 48 h, 72 h and 7 days after injection.

#### Image reconstruction and analysis of PET imaging data

The spatial resolution of the PET scanner is 1.5 mm (FWHM). All the images were reconstructed using a 2D OSEM iterative algorithm. The volume of interest (VOI) corresponding to significant uptake in each organ of interest (kidneys, liver, left ventricle, muscle, bone, brain) was delineated in the images using pMOD software. VOIs for liver and kidneys were determined by using thresholds in correlation with CT images. Image-derived input functions (blood kinetics) from the left ventricle were measured from PET images with computed tomography (CT)-based attenuation correction for all the C57/BL6 mice. Corresponding time activity curves (TACs) from each VOI were generated in order to determine the distribution kinetics of ^89^Zr-DFO-MQ1. The TACS are expressed as a percentage of the injected dose per volume (% ID/cc). From the TACs, AUC from time 0 to 7 days was calculated to define MQ1 uptake using Prism (Graph Pad software Inc., San Diego, USA).

The transfer rate from blood to kidneys or liver (*K_uptake,kidneys or Liver_*) was calculated by the integration plot method using the portion of time profile of the radioactivity during which radiotracer excretion from the organs back to the blood pool was negligible (from 15 s to 5 min). All statistical analyses were performed using PRISM software. Statistically significant differences in the data were determined using an unpaired Student's *t* test. Changes at the 95% confidence level (*P* < 0.05) were qualified as statistically significant.

### Confocal microscopy imaging

CHO and LLC-pk1 cells were from ATCC and cultured in DMEM medium (Life Technologies) supplemented with 10% FCS, 1% penicillin/streptomycin, 2 mM glutamine and 1% non-essential amino acids. CHO cells stably expressing human vasopressin and oxytocin receptors and rat oxytocin receptors were prepared in the laboratory and cultured in the same medium supplemented with 0.4 mg/mL G418. Human kidney carcinoma cells A-498 (ATCC-HTB-44) were from ATCC and were cultured in DMEM medium supplemented with 10% FBS, 1% penicillin/streptomycin and 1% non-essential amino acids. Human renal adenocarcinoma ACHN cells from ECACC (Sigma) were cultured in the same medium. Human Caucasian kidney carcinoma CAKI-2 cells from ECACC (Sigma) were cultured in McCoy's 5a supplemented with 10% FBS and 1% penicillin/streptomycin.

Freshly dissociated CHO, LLC-PK1 or renal cancer cell lines expressing vasopressin receptors were seeded on 12-mm glass coverslips pre-coated with poly-ornithine, cultured for 48 h before incubation in DMEM, 0.2 mg/mL BSA, 25 mM HEPES, pH 7.4 for 1 h at 12°C or 30 min at 22 °C with fluorescent analogues in the absence or presence of non-fluorescent ligands (30-min pre-incubation). After 3 washes with cold PBS, the cells were finally fixed in 4% PFA overnight at 4 °C and mounted with Mowiol. The fluorescent cells were imaged using a Zeiss LSM510 Meta confocal microscope equipped with a ×63 (NA 1.4) oil immersion objective. For excitation, available laser rays were 488 and 633 nm and emission signals were collected in multitracking mode with appropriate band pass filters. Labeled and unlabeled cells were localized by phase contrast microscopy and by using a 405 nm diode for imaging nuclei labeled with Hoechst.

## Supplementary Material

Supplementary figures and tables.

Movie 1.

## Figures and Tables

**Table 1:** Urinary parameters (24-h periods) in Sprague-Dawley rats after a single i.p. injection of various MQ1 doses. Statistical analysis by a post-hoc Dunnett test, with vehicle as standard. n: number of rats

	Dose (nmol/kg)	N	Diuresis (mL/h/kg)			Osmolarity (mOsmol/kgH_2_O)			Excretion (µosm/h/kg)		
			Average	Min-max.	*p* value	Averag	Min-max.	*p* value	Average	Min-max.	*p* value
0-24 h	Vehicle	22	1.56	0.72-2.59	-	1228	644-1913	-	1804	1234-2930	-
	1	6	2.34	1.52-3.90	1.0	1158	594-1480	0.69	2206	1644-2564	0.72
	3	6	5.13	3.21-11.68	0.02	503	277-594	<0.001	2710	1828-3226	0.003
	10	11	7.82	6.63-11.55	<0.001	273	195-387	<0.001	2092	1585-2795	0.38
	30	11	10.45	9.17-14.54	<0.001	214	154-275	<0.001	2280	1900-3082	0.005
	100	12	12.83	8.31-21.54	<0.001	162	98-224	<0.001	2046	1713-2234	0.97
	356	17	20.95	8.78-29.48	<0.001	136	80-250	<0.001	2658	2129-3364	<0.001
24 - 48 h	Vehicle	22	1.49	0.86-3.23	-	1290	648-1936	-	1652	966-3535	-
	3	6	1.53	1.36-6.40	0.93	2000	548-2220	0.008	3077	2285-3677	<0.001
	10	11	1.58	0.76-2.36	1.00	1332	1032-2020	0.91	2117	1510-2709	0.67
	30	11	2.25	1.78-3.37	0.75	937	688-1312	0.04	2064	1598-3091	0.01
	100	12	2.74	2.10-4.07	0.37	703	454-948	<0.001	1930	1599-2298	0.97
	356	17	9.64	3.32-19.50	<0.001	283	129-856	<0.001	2617	1721-3738	<0.001
72 h	Vehicle	17	1.3	0.83-2.61	-	1472	704-2096	-	1887	1446-2841	-
	356	12	6.00	2.05-7.37	<0.001	455	258-1420	<0.001	2617	1798-4216	0.01
96 h	Vehicle	16	1.42	0.86-2.54	-	1512	968-1812	-	1736	1338-2939	-
	356	12	3.76	2.74-5.23	<0.001	754	544-1252	<0.001	2838	2370-3600	<0.001

**Table 2:** Pharmacokinetics of MQ1 (356 nmol/kg, 2.27 mg/kg). n = 3, Route: i.p., Values are geometric means except for Tmax [median (min-max)] and t1/2 (harmonic mean). AUCt, area under the curve from time zero to the last sampling time; CL/F, apparent clearance; Vss/F, apparent volume of distribution at steady state; T1/2α and T1/2β, elimination half-time

PK parameters (unit)	Cmax (µg/mL)	Tmax (h)	AUClast (µg/mL*h)	Kel (h^-1^)	T_1/2α_ (h)	T_1/2β_ (h)	Clearance/F (mL/min/kg)	Vss/F (L.kg)
MQ1	2.10	4 (1-6)	11.6	0.0142	3.8	31	2.12	0.695

**Table 3:** Pharmacokinetic parameters describing ^89^Zr-DFO-MQ1 kinetics in blood and kidneys. T_1/2α_ is the fast contribution and the T_1/2β_ is the slow contribution

	T_1/2α_ Blood (h)	T_1/2β_ Blood (h)	T_1/2α_ Kidneys (h)	T_1/2β_ Kidneys (h)	AUC_blood_ (% ID/CC × h)	AUC_liver_ (% ID/CC × h)	AUC_Kidneys_ (% ID/CC × h)	k_uptake,liver_ (mL/min/g)	K_uptake,kidneys_ (mL/min/g)
MQ1	1.4±0.6	26±15	0.9±0.4	46±11	36±4.3	820±52	1850±121	0.32±0.16	0.53±0.19

**Table 4:** Diuresis and urine osmolality over a 24-h period in Sprague-Dawley rats injected daily i.p. with 3 nmol/kg of MQ1. N = 22 for vehicle and 5 for MQ1-treated rats. P values compared with vehicle

Days	Diuresis (ml/h/kg)			Osmolarity (mOsm/kg H_2_O)			Excretion (µOsm/h/kg)		
	Average	Min-max.	*p* value	Average	Min-max.	*p* value	Average	Min-max.	*p* value
Vehicle	1.44	0.72-3.23	-	1375	644-2096	-	1770	966-3535	-
1	6.3	4.8-7.0	<0.001	328	285-359	<0.001	1919	1601-2100	0.41
2	5.9	5.1-7.2	<0.001	317	296-366	<0.001	1820	1499-2399	0.85
3	6.00	4.98-6.40	<0.001	313	232-396	<0.001	1805	1394-2157	0.92
4	5.7	5.34-6.47	<0.001	326	266-389	<0.001	1940	1600-2179	0.26
Average	6.0			321			1871		

## Abbreviations

ADPKD: autosomal dominant form of polycystic kidney disease
AVP: arginine-vasopressin
PD: pharmacodynamics
PET: positron emission tomography
PK: pharmacokinetics
V2R: type 2 vasopressin receptor
