# Supplementary material for: A snake toxin as a theranostic agent for the type 2 vasopressin receptor
Source: Theranostics. 2020 Sep 18;10(25):11580–94. doi: 10.7150/thno.47485 (PMC7545998; doi:10.7150/thno.47485)
Supplement: Supplementary file 1 — Supplementary figures and tables. [file thnov10p11580s1.pdf]

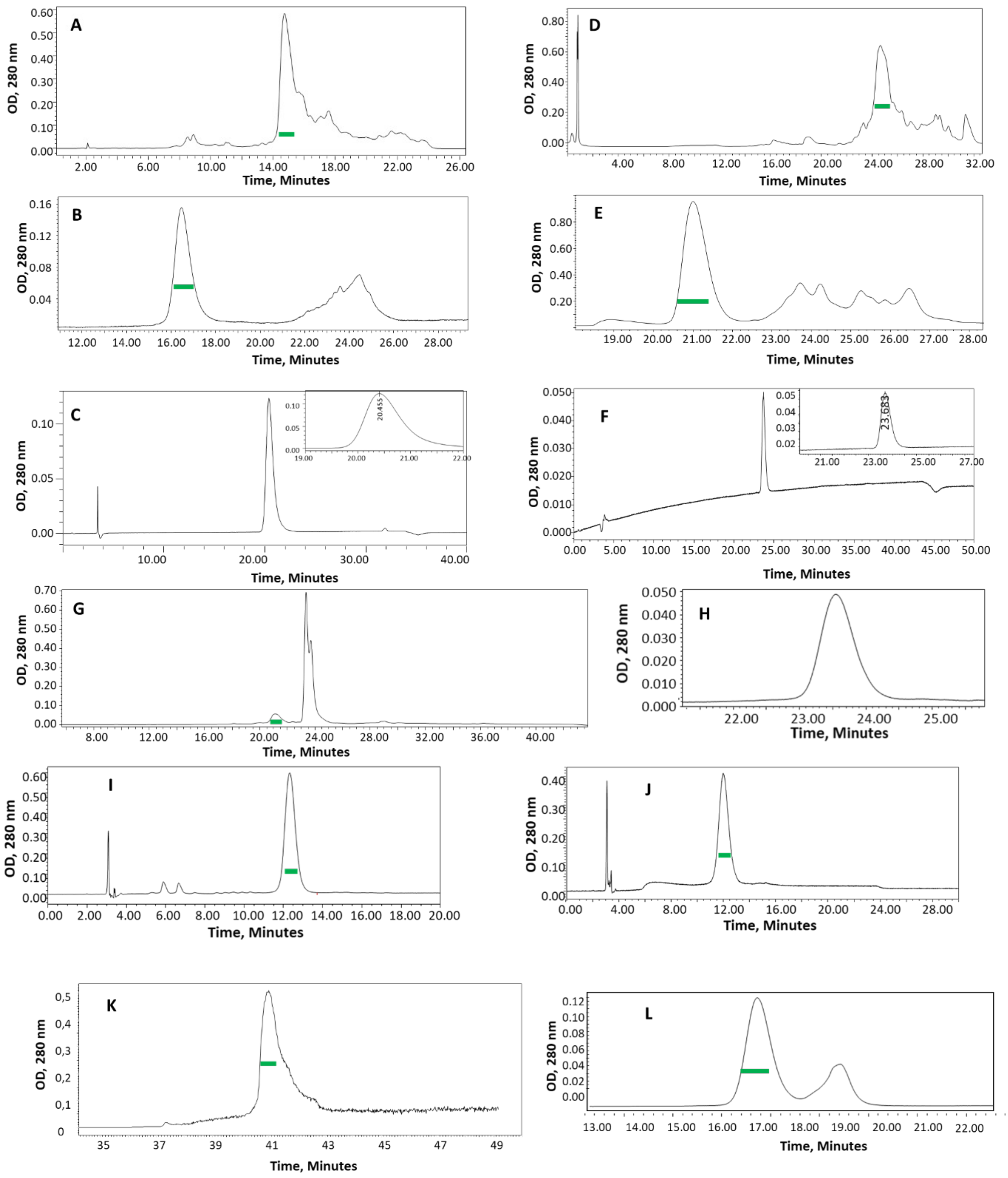

Figure S1. Chemical synthesis of MQs. **(A)** Reverse-phase liquid chromatography of the crude synthesis of MQ1 on Waters X-bridge C18, 19 × 250 mm, 10 µm, flow of 15 mL/min. The gradient was 10 to 40% of solvent B in 30 min. **(B)** Reverse-phase liquid chromatography of crude oxidized MQ1 on a Waters Sunfire 10 µm, 10 × 250 mm, 5 mL/min, same gradient. **(C)** Analytical chromatography of the pure oxidized MQ1 performed on Waters X-bridge C18, 4.6 × 150 mm, 3.5 µm. The gradients were 10 to 50% of solvent B in 40 min. Insert peak zoom. **(D)** Reverse-phase liquid chromatography of the crude synthesis of 6-azidohexanoic-MQ1 on Waters X-bridge C18, 19 × 250 mm, 10 µm, flow of 15 mL/min. The gradient was 10 to 40% of solvent B in 30 min. **(E)** Reverse-phase liquid chromatography of crude oxidized 6-azidohexanoic-MQ1 on a Waters Sunfire 10 µm, 10 × 250 mm, 5 mL/min, same gradient. **(F)** Analytical chromatography of the pure oxidized MQ1 performed on Waters X-bridge C18, 4.6 × 150 mm, 3.5 µm. The gradient was 10 to 50% of

solvent B in 40 min. Insert peak zoom. **(G)** Reverse-phase liquid chromatography of deferoxamine-DBCO-MQ1 on Waters X-bridge C18, 4.6 × 150 mm, 3.5 µm. The gradient was 20 to 60% of solvent B in 40 min. **(H)** Zoom of the analytical chromatography of the pure deferoxamine-DBCO-MQ1 performed on Waters X-bridge C18, 4.6 × 150 mm, 3.5 µm. Reverse-phase liquid chromatography of AFDye-488-DBCO-MQ1 **(I)** or Cy5.5-DBCO-MQ1 **(J)** on Waters X-bridge C18, 4.6 × 150 mm, 3.5 µm. The gradient was 30 to 60% of solvent B in 30 min. solvents are A (H_2O, TFA 0.1%) and B (acetonitrile, TFA 0.1%). Color bar indicates collected fractions. **(K)** Reverse-phase liquid chromatography of the crude synthesis of MQ1 variant on Waters X-bridge C18, 19 × 250 mm, 10 µm, flow of 15 mL/min. The gradient was 10 to 40% of solvent B in 30 min. **(L)** Reverse-phase liquid chromatography of crude oxidized MQ1 variant on a Waters Sunfire 10 µm, 10 × 250 mm, 5 mL/min, same gradient.

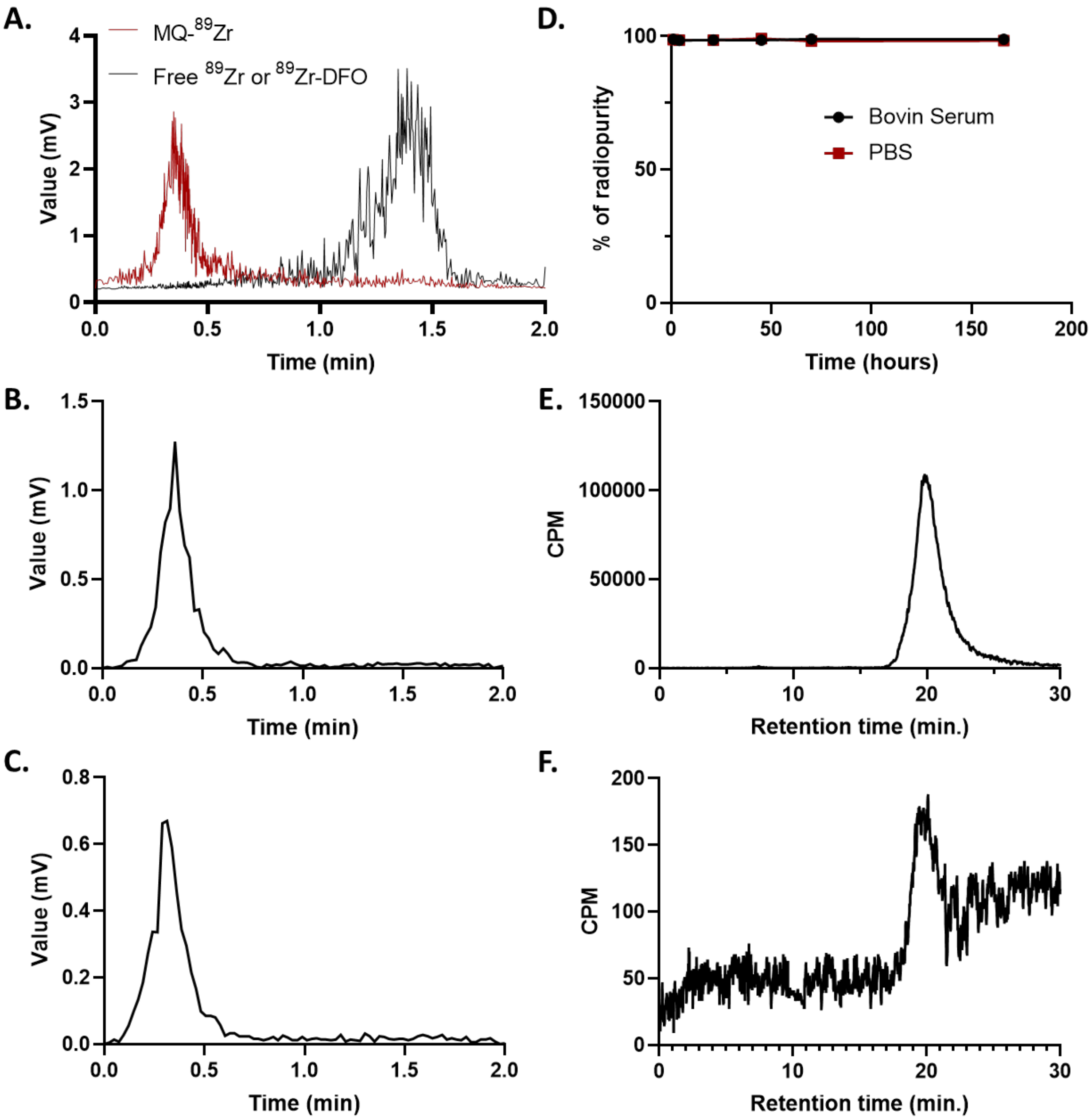

Figure S2. **(A)** Representative iTLC chromatograms of fresh ^{89}Zr-DFO-MQ1 and free ^{89}Zr. iTLC chromatograms of blood sampling 1.5 h after injection (**B**) and 7 days after injection (**C**). **(D)** *In vitro* stability of ^{89}Zr-DFO-MQ1 in bovine serum and PBS determined by iTLC. **(E)** RadioHPLC characterization of fresh ^{89}Zr-DFO-MQ1. **(F)** RadioHPLC characterization of blood sampling at 7 days post injection.

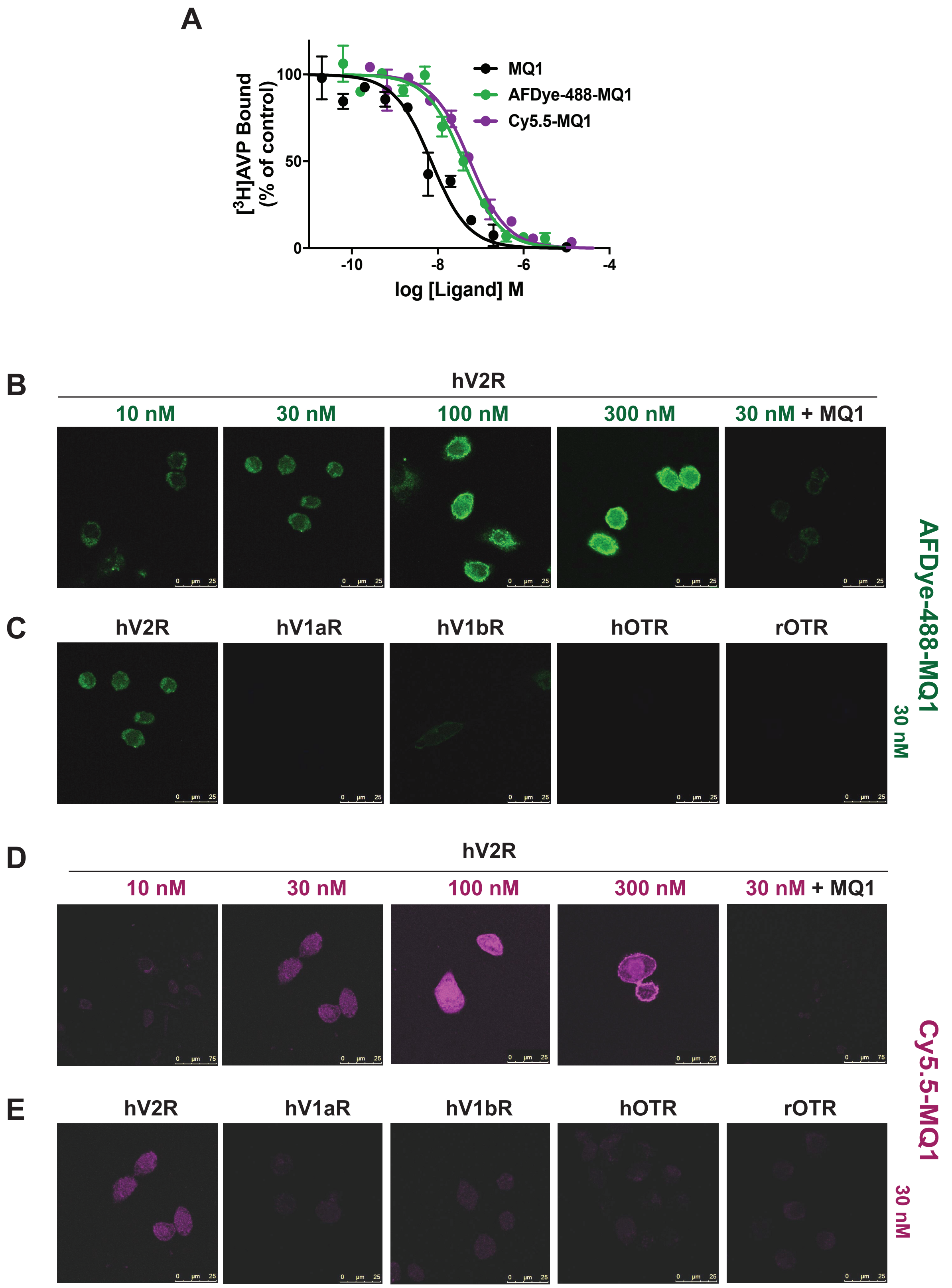

Figure S3. **(A)** Binding curve on hV2R of MQ1, Cy5.5-MQ1 and AFDye-488-MQ1. **B)** Labeling of hV2R expressed in stable CHO cell line with increasing doses of AFDye-488-MQ1. Control without AFDye-488-MQ1 looks like the 10 nM cell labeling. Specificity is determined by the last panel in presence of 3.4 μM MQ1. **(C)** Selectivity of 30 nM AFDye-488-MQ1 to hV2R labeling compared to hV1aR, hV1bR, hOTR and rOTR expressed stably in CHO cell lines. **(D)** and **(E)** are the same than **(B)** and **(C)** but using the Cy5.5-MQ1 probe.

Table S1: Blood sample preparation for PK determination

**Sample**			
Rat plasma	100 µL of rat plasma		
Dilution	400 µL of TFA 0.1%		
**Solid Phase Extraction Oasis HLB 1 cc 30 mg cartridge (Waters) / SpeedDisk (Biotage)**			
Deposit	500 µL		
Wash	2 x 500 µL H_2O/MeOH (9/1) + TFA 0.1%		
Elution	2 x 150 µL H_2O/MeOH (2/8) + TFA 0.1%		
Evaporation	to dryness under N_2 at 40°C		
**Reduction/Alkylation**			
Reduction	25 µL DTT 100 mM (in AB 50 mM)-incubation 10 min at 90°C.		
Alkylation	25 µL IAA 225 mM (in AB 50 mM)-incubation 30 min at RT to darkness		
Addition	10 µl H_2O/ACN (9/1) 0.5% HCOOH		
**Centrifugation**	20 000 g /15 min		
**Vial transfer**	40 µL		
**Injection**	10 µL into the UPLC-MS/MS system		
NB:			
TFA	trifluoroacetic acid	AB	ammonium bicarbonate
MeOH	methanol	IAA	iodoacetamide
DTT	dithiothreitol	RT	room temperature
H_2O	water	ACN	acetonitrile

Table S2: parent ion > product ion transition monitored

	Transitions monitored	Role
MQ1	**673.03 > 731.62**	**quantification**
	673.03 > 775.09<br>747.58 > 836.10<br>841.02 > 975.05	specificity
MQ1 variant	**666.97 > 735.39**	**Used for internal standard (IS) normalization**
	740.87 > 840.60	IS specificity

Table S3. PK values and PK PD concentrations under modeling.

Times (h)	**MQ1 plasma concentrations µg/ml**	**Plasma concentration with modelling (µg/mL)**	**Diuresis values with modelling (mL/h/kg)**
1	1.709	1.305	27.69
2	0.486	0.783	80.74
3	0.259	0.606	55.34
4	1.239	0.469	37.93
5	0.257	0.363	26.00
6	0.378	0.281	17.82
8	0.150	0.169	14.79
10	0.299	0.178	14.12
12	0.219	0.173	13.48
16	0.154	0.164	12.28
24	0.096	0.148	10.20
48	0.056	0.108	5.83
72	0.092	0.079	3.34
96	0.074	0.058	1.91
